# Editorial: Application of Nanotechnology in Food Science and Food Microbiology

**DOI:** 10.3389/fmicb.2018.00714

**Published:** 2018-04-18

**Authors:** Jayanta Kumar Patra, Han-Seung Shin, Spiros Paramithiotis

**Affiliations:** ^1^Research Institute of Biotechnology and Medical Converged Science, Dongguk University-Seoul, Goyangsi, South Korea; ^2^Department of Food Science and Biotechnology, Dongguk University-Seoul, Goyangsi, South Korea; ^3^Department of Food Science and Human Nutrition, Agricultural University of Athens, Athens, Greece

**Keywords:** nanotechnology, nanomaterials, processing aids, quality enhancers, antimicrobial agents, biosensors, sustainability

Nanotechnology is a rapidly evolving field with potential applications that extend over a wide range of scientific disciplines. In Food Science and Technology, nanomaterials have been effectively used as processing aids, quality enhancers, antimicrobial agents as well as in the development of sensors for quality and safety assessment. The increased interest for their use has led to the development of a wide variety of synthesis approaches and protocols, each offering specific advantages and suffering from specific limitations. On the other hand, there are several concerns regarding their possible negative effects on the environment and human health. This Research Topic consists of 17 articles that provide with recent advances and insights within the above mentioned fields.

Nanotechnology has provided with alternative approaches in food processing regarding both improvement of the physicochemical properties and enhancement of nutrient stability and bioavailability of the product. In this research topic these issues are addressed in five manuscripts. A comprehensive literature review on the current advancements in liposome manufacturing and applications in food and pharma sectors was offered by Shukla et al. An insight on the interactions between silica dioxide nanoparticles and food components was provided by Go et al. It was calculated that on the silica dioxide nanoparticles only a small percentage of the food carbohydrates, fatty acids, minerals, and proteins was adsorbed. In addition, it was indicated that other minor food constituents could also interact with the nanoparticles. In conclusion, the rather restricted extend of interactions could not affect the solubility of the nanoparticles. An evaluation of the novel strategy in food fortification employing surface modifications was offered by Kim et al. Surface modification is employed to address the disadvantages of fortification that are associated with unacceptable changes in the sensorial properties. In this study, two products that make use of such modifications, namely SunActive Zn™ and SunActive Fe™ were evaluated in terms of bioavailability and potential toxicity. Regarding both products, enhanced cellular uptake, intestinal transport efficacy, and bioavailability were reported, without any affecting potential cytotoxicity. Dahiya et al. discussed the factors that define the interplay between gut microbiota and host energy homeostasis in the context of obesity along with therapeutic approaches mostly through the pro- and pre- biotic concept as indicated by Yadav et al. as well.

The antimicrobial effect of a wide range of nanoparticles has been given considerable attention and a series of applications, mainly as far as food contact surfaces and food packaging are concerned, have been proposed. The antimicrobial activity of cationic nanostructures prepared from a combination of the cationic lipid dioctadecyldimethylammonium bromide (DODAB), the antimicrobial peptide gramicidin D (Gr), the antimicrobial cationic polymer poly(diallyldimethylammoniumchloride) (PDDA), and the biocompatible polymer poly(methylmethacrylate) (PMMA) against *Escherichia coli, Salmonella* Typhimurium, *Staphylococcus aureus* and *Listeria monocytogenes* in 1 mM NaCl was assessed by Carrasco et al. The reported data revealed the different sensitivity of the studied microorganisms to the compounds examined. More accurately, PMMA/PDDA was the most effective nanostructure against *S*. Typhimurium and *St. aureus*. On the other hand, DODAB BF and DODAB BF/Gr were the most effective nanostructures against *E. coli* and *L. monocytogenes*, respectively. On the contrary, Gr was the least effective against *E. coli* and *St. aureus*, PMMA/DODAB against *S*. Typhimurium and PDDA against *L. monocytogenes*. In the study of Gkana et al. commercially available organosilane based products were applied on glass and stainless steel surfaces and then the ability of *L. monocytogenes, S*. Typhimurium, *E. coli* O157:H7, *St. aureus* and *Yersinia enterocolitica* to form biofilms was assessed. It was demonstrated that the anti-adhesive and anti-biofilm activity of the specific products was highly dependent upon the surface type, the microbial species and the incubation time.

The development of biosensors for the detection of both chemical and biological contaminants, through the utilization of the unique properties that the nanomaterials offer, is an intensively studied and actively growing field. Within the frame of this research topic, Wang et al. and Oliveira et al. reported the development of two reliable tools. In the former case, Wang et al. reported the development of a biosensor for the detection of *Shigella* spp. that combined multiple cross displacement amplification (MCDA) with gold nanoparticle-based immunochromatographic detection. MCDA is a newly established isothermal amplification approach that promises to address the limitations of the existing isothermal amplification strategies. On the other hand, gold nanoparticles have been extensively employed in biosensor development due to their electronic and optical properties as well as their biocompatibility. The entire analysis, from sample preparation to amplicon detection was reported to be completed within 1 h. The analytical sensitivity was determined in pure cultures to 10 fg of genomic template per reaction and 5.86 CFU per tube in human fecal samples. In the case of Oliveira et al., the development of a reliable tool for the detection and enumeration of released lactococcal prophages based on flow cytometry following Mitomycin-C treatment was reported. Several flow cytometry-based techniques have been developed over the recent years as an alternative approach to the classical plaque assay, offering speed, sensitivity, and reproducibility. On the other hand, Mitomycin-C is a well-known antitumor antibiotic that is used in prophage viability assessment since it induces the lytic cycle. The effectiveness of this approach was exhibited since the results obtained with this approach and transmission electron microscopy were in concordance.

Nanoparticle synthesis approaches may be distinguished according to the size of the starting material into the top-bottom and the bottom-up ones and according to their nature to chemical, physical, and biological ones. The latter have been the epicenter of intensive study over the last decade in an attempt to address the current trend toward the use of eco-friendly strategies and to reduce the use of hazardous chemicals. Diatoms are unicellular algae that are involved in the biogeochemical silica cycling; therefore their use in nanobiotechnological applications has attracted significant attention generating a wealth of literature. Mishra et al. reviewed this literature and presented the applicability of diatoms in nanotechnology and biotechnology in a concise and comprehensive way. The synthesis of silver nanoparticles using the aqueous extract of *Zea mays* leaf waste, a waste material from the corn industry, was reported by Patra and Baek. The antimicrobial activity of the produced nanoparticles as such or in combination with standard antimicrobial agents was evaluated against *Bacillus cereus, L. monocytogenes, St. aureus, E. coli, S*. Typhimurium, *Candida albicans, C. glabrata, C. geochares*, and *C. saitoana*. The nanoparticles exhibited considerable antibacterial activity that was further enhanced upon combination with kanamycin and rifampicin. On the other hand, no anticandidal activity was observed upon application of 50 ug/mL of silver nanoparticles. Potent anticandidal activity was observed when the silver nanoparticles were combined with amphotericin b. Liu et al. provided with a critical insight regarding the huge potential for biocombinational synthesis provided by the nonribosomal peptide synthetases (NRPSs) and demonstrated the synthesis of five novel lipopeptides through reprogramming of the plipastatin biosynthetic machinery. More accurately, two cyclic products, namely cyclic pentapeptide and octapeptide and three linear ones, namely hexapeptide, nonapeptide, and heptapeptide were identified. The latter exhibited strong antifungal activity against *Rhizopus stolonifer, Fusarium oxysporum, Aspergillus ochraceus, A. niger*, and *Penicillium notatum* as well as notable antibacterial activity against *E. coli* and *St. aureus*. Bhardwaj et al. decorated cotton fibers with silver nanoparticles using a simple, rapid, eco-friendly, and cost effective approach. Then, the antibacterial activity of the composite against *E. coli, St. aureus*, and *S*. Typhimurium was demonstrated through both disk diffusion and broth microdilution assays. The results indicated the suitability of this composite as a surface disinfectant for a variety of applications.

The rapid development of novel nanomaterials and related applications is followed by several concerns regarding their effect on human health and the environment. An overview of the recent developments, challenges and perspectives of nanotechnology within the frame of sustainable agriculture was offered by Prasad et al. A focus on the toxicity of silver nanoparticles and their effect on lower and higher autotrophic plants as well as heterotrophic microorganisms along with the mode of toxicity and the tolerance mechanisms was provided by Tripathi et al. In addition, a comparative study on the toxicity of silver nanoparticles and silver nitrate on hydroponically grown mustard (*Brassica* sp.) seedlings was reported by Vishwakarma et al. Both compounds affected negatively the indices employed, namely shoot and root length and fresh mass, total chlorophyll and carotenoids concentration, total protein content, ascorbate peroxidase and catalase activities, oxidative damage, DNA degradation, accumulation of the compounds and cell viability. In all cases the effect of silver nitrate was more pronounced than the respective of silver nanoparticles. Finally, Singhal et al. studied the effect of a novel nanotool resulting from the association of zinc oxide nanorods and the fungus *Piriformospora indica*, named nano-embedded fungus, on *Brassica oleracea* var *botrytis* growth. It was reported that this novel nanotool enhanced the crop productivity by improving seed germination rate, shoot and root length as well as fresh and dry weight.

This research topic contains a collection of articles that address all aspects of the application of nanotechnology in food science and food microbiology. The editors believe that through this series of articles, the current range of applications along with their limitations has been adequately highlighted, facilitating the identification of knowledge gaps and research opportunities.

## Author contributions

JKP and SP: wrote the editorial, JKP, SP, and H-SS: edited the editorial. All the authors read and approved the editorial.

### Conflict of interest statement

The authors declare that the research was conducted in the absence of any commercial or financial relationships that could be construed as a potential conflict of interest.

